# A Neuro-Fuzzy System for Characterization of Arm Movements

**DOI:** 10.3390/s130202613

**Published:** 2013-02-21

**Authors:** Alexandre Balbinot, Gabriela Favieiro

**Affiliations:** Electrical Engineering, Instrumentation Laboratory, Federal University of Rio Grande do Sul, Avenue Osvaldo Aranha 103, Porto Alegre 90035-190, Brazil; E-Mail: aulaeng@gmail.com

**Keywords:** biomedical instrumentation, surface electromyography (sEMG), arm movements, neuro-fuzzy system

## Abstract

The myoelectric signal reflects the electrical activity of skeletal muscles and contains information about the structure and function of the muscles which make different parts of the body move. Advances in engineering have extended electromyography beyond the traditional diagnostic applications to also include applications in diverse areas such as rehabilitation, movement analysis and myoelectric control of prosthesis. This paper aims to study and develop a system that uses myoelectric signals, acquired by surface electrodes, to characterize certain movements of the human arm. To recognize certain hand-arm segment movements, was developed an algorithm for pattern recognition technique based on neuro-fuzzy, representing the core of this research. This algorithm has as input the preprocessed myoelectric signal, to disclosed specific characteristics of the signal, and as output the performed movement. The average accuracy obtained was 86% to 7 distinct movements in tests of long duration (about three hours).

## 1. Introduction

Research in Biomedical Engineering and Computational Intelligence is providing mechanisms to help people with some disabilities to perform simple tasks of day-to-day. Studies in this area are justified by the fact that approximately 15% of the World population has some type of disability [1]. Because of physical disability, a significant portion of society has some personal limitations, therefore also for the professional and social life. According to Schulz *et al.* [2] in the United States there 41,000 registered persons who have had an amputation of a hand or a complete arm. With the same frequency of occurrence (1 in 6,100) there would be 1,000,000 such persons worldwide. Recent advances in signal processing technologies and mathematical models have made it practical to develop advanced EMG detection and analysis techniques. In 2006 various mathematical techniques and Artificial Intelligence (AI) have received extensive attraction [3]. In recent years, there has been an explosion of interest in Computational Intelligence as evidenced by the numerous applications in health, biomedicine, and biomedical engineering [4–15]. Computational Intelligence techniques are computing algorithms and learning machines, including artificial neural networks, fuzzy logic, genetic algorithms, and support vector machines.

The human skeletal muscular system is primarily responsible for providing the forces required to perform various actions. Currently, electromyography (EMG) studies are used for evaluating and diagnosing patients with neuromuscular disorders. The interpretation of EMG readings is usually performed by trained person. Problems arise when there are too few experts to meet the demand of patients and, therefore, it is becoming increasingly important to developed automated diagnostic systems based on EMG readings. This need provides scope for the application of Computational Intelligence techniques for the detection and classification of neuromuscular disorders based on EMG processing. The myoelectric signal is the sign of muscle control of the human body that contains the information of the user's intent to contract a muscle and, therefore, make a move. It makes the use of this signal very advantageous, because the control of a robotic prosthesis can be performed based in the intention of the user.

The work of Ahsan *et al.* [16] represents the detection of four hand motions (left, right, up and down) using an artificial neural network (ANN). According to Hudgins *et al.* [17], the success of a pattern classification system depends completely on the choice of features used to represent the raw EMG signals. Ahsan *et al.* [16] used seven statistical time and time-frequency based features: mean absolute value, root mean square, variance, standard deviation, zero crossing, slope sign change and Wilson amplitude.

Rajesh *et al.* [18], used wavelets and classification using Euclidean distance. The general features of the hand gestures from EMG signal patterns were: hand extension, hand grasp, wrist extension, wrist flexion, and pinch and thumb flexion. They used the entropy, rms and standard deviation in the analysis of features. Shenoy *et al.* [19], used a simple feature (rms value over windows) and continuously classified windows of data collected while the subject maintained a static hand gesture (gestures correspond to pairs of actions: grasp-release, left-right, up-down and rotate). The classification was with Linear Support Vector Machines. Boschmann *et al.* [20], introduce an approach for classifying EMG signals taken from forearm muscles using support vector machines. In a single experiment run, the test subject had to perform a sequence of eleven different movements: extension, flexion, ulnar deviation, radial deviation, pronation, and supination, open, close, key grip, pincer grip and extract the index finger.

Matrone *et al.* [21] presents the work of a robotic hand employing a two differential channels (four electrodes) EMG acquisition system and a principal components analysis (PCA) based controller. Participants volunteered in experimental tasks consisting in grasping, transporting and releasing different kinds of objects, by employing a five-fingered (and six motors) robotic hand. The control system decoded and converted the subjects' 2-DoF wrist contractions (flexion/extension and adduction/abduction) into hand posture control commands, implementing the algorithm based on PCA [22].

Within the last dozen of years, different structures of neuro-fuzzy networks have been presented, often referred to in the world literature as neuro-fuzzy systems [23]. They combine the advantages of neural networks and fuzzy systems. Kurzynski *et al.* [24], used the following methods of sequential classification (five types): Bayes approach with Markov model, multilayer perceptron, multiclassifier with competence function, classifier based on fuzzy logic and classifier based on Dempster-Shafer theory of evidence. In this paper it is proposed a method to determine the input features based on autoregressive (AR) model. Six different types of grapes depending on the grasping object (a pen, a credit card, a computer mouse, a cell phone, a kettle and a tube) were chosen for recognition. Seven elementary actions were distinguished in the process of grasping with a hand: rest position, grasp preparation, grasp closing, grabbing, maintaining the grasp, releasing the grasp, transition to the rest position. The paper of George *et al.* [25], concentrates on the classification of different speeds of movement of human elbow. For this, EMG signals are acquired from the *biceps brachii*. Two types of classifiers are developed and compared: Fuzzy Logic Classifier (FLC) and Probabilistic Neural Network Classifier (PNNC). Khezri *et al.* [26] propose to use an adaptive neuro-fuzzy inference system (ANFIS) to identify hand motion commands (hand opening and closing, pinch and thumb flexion, wrist flexion and extension), but with vision feedback to increase the capability of the system. In this work a hybrid method for training fuzzy system, consisting of back-propagation (BP) and least mean square (LMS) was utilized. In this study was used, mean absolute value, slope sign changes and AR model coefficients as time features of the signal. Khezri *et al.* [27], used two classifiers: fuzzy inference system (FIS) and artificial neural network (ANN). They consider four major parts including sEMG preprocessing, and conditioning, feature extraction (time domain, time-frequency domain and their combination), dimensionality reduction [applied to simplify the task of the classifier: two approaches: class separability (CS) and principle component analysis (PCA)] and classification. Eight hand movements were extracted: hand opening and closing, pinch, thumb flexion, wrist radial flexion and extension and wrist flexion and extension.

**Figure 1.**
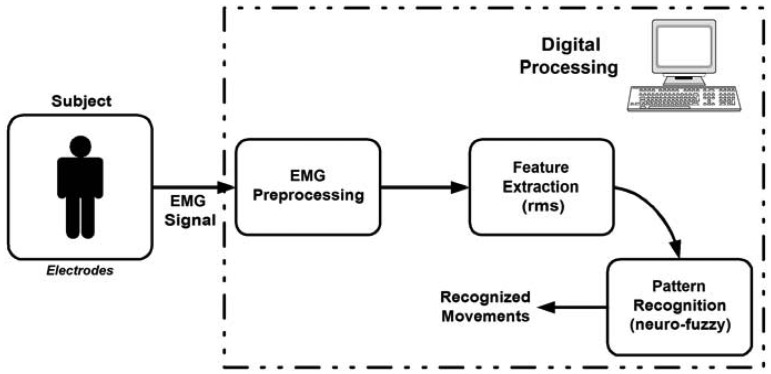
A block-diagram representation of the system.

Therefore, it is possible to distinguish certain muscle movements while processing the electrical parameters of the myoelectric signal. Considering that premise, this research aims to study and develop a system that uses myoelectric signals, acquired by surface electrodes, to characterize certain movements of the human arm. The pattern recognition system (see Figure 1) has three basic components: preprocessing (filtering, calibration of each channel, removal of DC component, windowing the signal of interest), the feature extraction (determining the rms value of the signal of interest) and classification (neuro-fuzzy). To recognize certain arm movements (see Table 1), an algorithm was developed for pattern recognition based on neuro-fuzzy logic, representing the core of this research. Fuzzy logic systems can emulate human decision-making more closely than many other classifiers, because of the possibility of introducing the knowledge of an expert system in the fuzzy rules [5,28]. The non-stationary nature of EMG signals, like other biological signals, makes the classification task more difficult, but the characteristics of a fuzzy inference system make it a viable tool for pattern recognition applications [29]. The algorithm developed has as input the preprocessed myoelectric signal, to disclosed specific characteristics of the signal, and as output the performed movement. The proposed system uses only eight pairs of electrodes for signal acquisition (without visual feedback as output), and processes these signals with a neuro-fuzzy system for recognition of performed movements. Therefore, in this work we applied a neuro-fuzzy method to recognize EMG patterns. Neuro-fuzzy computing enables us to build a more robust intelligence-based decision making system by combining the advantages of artificial neural networks with the fuzzy modeling of imprecise and qualitative knowledge.

## 2. Sugeno Inference System and Adaptive Neuro-Fuzzy: An Introduction

A fuzzy set is defined as a set or collection of elements with membership values between 0 and 1. Therefore, the transition between belonging or not belonging to the set is gradual and is characterized by its fuzzy Membership Function (MF) that is used to describe the fuzzy membership value given to fuzzy set elements enabling the fuzzy set model linguist expression used in everyday life, such as, “the rms value of the masseter myoelectric signal is medium high”.

- For this study the Sugeno fuzzy model had been used to generate fuzzy rules from a set of input and outputs. A typical fuzzy rule in the Sugeno fuzzy model is shown in Equation (1):
- If *x* is equal to *A* and *y* is equal to *B*, then:
  (1) z=f(x,y)

as *A* and *B* sets of fuzzy antecedents and *z* = *f*(*x*,*y*) the crisp consecutive function.

Adaptive Neuro-Fuzzy Inference System (ANFIS) is a class of adaptive networks whose functionality is equivalent to a fuzzy inference system (FIS), which generates a fuzzy rule and membership functions (MF) automatically. The output of this system can be described by the following Equation (2):

(2) $$\gamma =\sum_{i=1}^{L}\left\{\frac{\left(\prod_{j=1}^{n}M{\text{F}}_{j}^{i}\left(x_{j}\right)\right)\left(z^{i}\right)}{\sum_{i=1}^{L}\left(\prod_{j=1}^{n}M{\text{F}}_{j}^{i}\left(x_{j}\right)\right)}\right\}$$

where MF is the membership function, *x_j_* (*j* = 1, 2, …, *n*) is the *j^th^* input and *z^i^* is the output of *j^th^* fuzzy rule. ANFIS adapts the parameters of Sugeno type inference system using the neural networks.

**Figure 2.**
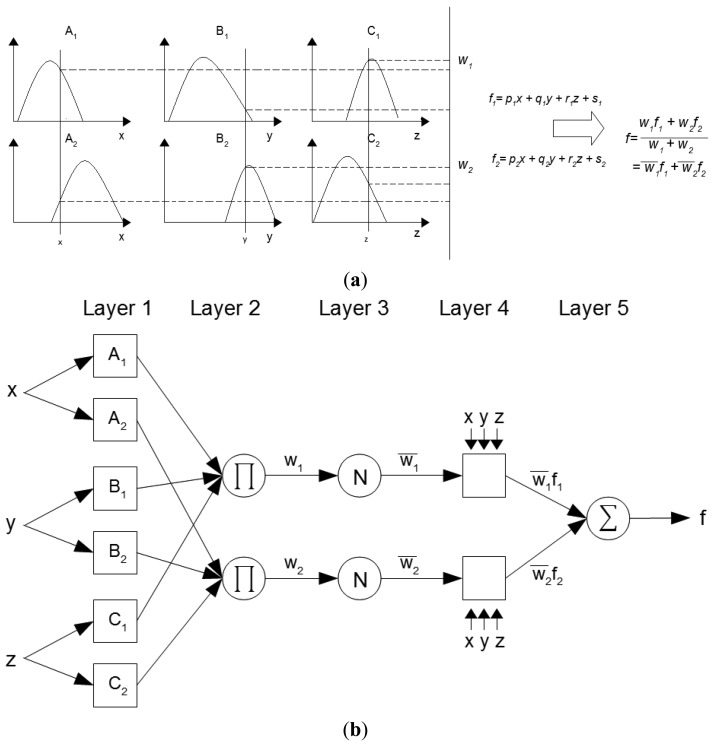
(**a**) Example of a Sugeno Inference Model: three inputs and two rules and (**b**) The equivalent ANFIS architecture.

Typically the Adaptive Neuro-Fuzzy Inference System network topology consists of connected nodes that depend on parameters that change according to certain learning rules that minimize the error. The learning technique most commonly used is the gradient method, however Jang [30] proposed hybrid learning rule which includes the Least Square or simply LSE Estimator. Considering a fuzzy system with three inputs *x*, *y* and *z*, one output *v* and a fuzzy inference Sugeno model. One possible set of rules is shown in Equations (3) and (4):

(3) $$\mathrm{Rule}\;1:\;If\;x\;is\;\mathrm{equal}\;to\;A_{1},\;y\;is\;\mathrm{equal}\;to\;B_{1},\;\mathrm{and}\;z\;is\;\mathrm{equal}\;to\;C_{1},\;\mathrm{then}\;f_{1}=\;p_{1}x\;+\;q_{1}y\;+\;r_{1}y\;+\;s_{1}$$

(4) $$\mathrm{Rule}\;2:\;If\;x\;is\;\mathrm{equal}\;to\;A_{2},\;y\;is\;\mathrm{equal}\;to\;B_{2},\;\mathrm{and}\;z\;is\;\mathrm{equal}\;to\;C_{2},\;\mathrm{then}\;f_{2}=\;p_{2}x\;+\;q_{2}y\;+\;r_{2}z\;+\;s_{2}$$

as an example, Figure 2(a) illustrates the reasoning mechanism for the Sugeno inference model. The equivalent ANFIS architecture is presented in Figure 2(b) with nodes of same layer having similar functions.

The first layer of Figure 2(b) is represented by adaptive nodes *i* whose functions are determined by Equations (5–7):

(5) $$O_{1,i}=\mu_{A_{i}}\left(x\right),\;\text{for}\;i=1,2$$

(6) $$\;O_{1,i}=\mu_{B_{i-2}}\left(y\right),\;\text{for}\;i=3,\;4$$

(7) $$\;O_{1,i}=\mu_{C_{i-4}}\left(y\right),\;\text{for}\;i=5,\;6$$

where *x, y or z* entries in node *i* and *A_i_B_i-2_* and *C_i-4_* linguistic labels associated with that node. Thus, *O_1,i_* represents the pertinence degree to the fuzzy set *A* (*A_1_*, *A_2_*, *B_1_*, *B_2_*, *C_1_* or *C_2_*) and specifies the degree to each input *x, y or z* satisfies the fuzzy set *A*. The membership function *μ* can be any of the membership functions: triangular membership function, Gaussian-membership function, Bell membership function, *etc*. When the values (called the premise parameters) of the membership function are changed, the function varies, *i.e.*, display various types of MF to the fuzzy set *A*. The layer 2 has fixed nodes indicated by *Π* with outputs that represents the input signals product, as indicated in Equation (8)—the output nodes represent the firing strength of a given rule:

(8) $$O_{2,1}=\omega_{i}=\mu_{A_{i}}\left(x\right)\mu_{B_{i}}\left(y\right)\mu_{C_{i}}\left(z\right),\;i=1,\;2.$$

In layer 3 the fixed nodes are referred to *N*. The *i_th_* node calculates the firing strength rate of rule *i_th_* to the sum of all firing strength of rules, given by Equation (9)—the nodes in layer 3 are generally known as normalized firing strength:

(9) $$O_{3,i}=\bar{\omega_{\iota }}=\frac{\omega_{i}}{\omega_{1}+\omega_{2}},\;i=1,\;2.$$

Layer 4, for example, the nodes *i* are adaptive with the function given by Equation (10):

(10) $$O_{4,i}=\bar{\omega_{\iota }}f_{i}=\bar{\omega_{\iota }}(p_{i}x+q_{i}y+r_{i}z+s_{i})$$

where $\bar{\omega_{\iota }}$ is a normalized firing strength from layer 3 and {*p_i_, q_i_, r_i_, s_i_*} the set of parameters (called consequence parameters) of this node. The last layer of the Figure 2(b) has only one fixed node called Σ that determines the final output as the sum of all signals represented by Equation (11):

(11) $$\text{Final Output}=\;O_{5,1}=\sum_{i}\bar{\omega_{\iota }}f_{i}=\;\frac{\sum_{i}\omega_{i}f_{i}}{\sum_{i}\omega_{i}}$$

Considering the architecture shown in Figure 2(b) it can be seen that while the values of the parameters of the premises is fixed, the final output can be expressed as a linear combination of consequence parameters. Therefore, the output can be rewritten, for example, by the linear equation with the following consequence parameters: *p_1_*, *q_1_*, *r_1_*, *s_1_, p_2_*, *q_2_*, *r_2_* and *s_2_* (see Equation (12)):

(12) $$\begin{array}{ccc} f & \begin{array}{c} =\\ =\\ = \end{array} & \begin{array}{c} \frac{\omega_{1}}{\omega_{1}+\omega_{2}}f_{1}+\frac{\omega_{2}}{\omega_{1}+\omega_{2}}f_{2}\\ \bar{\omega_{\iota }}\left(p_{1}x+q_{1}y+r_{1}z+s_{1}\right)+\;\bar{\omega_{2}}\left(p_{2}x+q_{2}y+r_{2}z+s_{2}\right)\\ \left(\bar{\omega_{\iota }}x\right)p_{1}+\left(\bar{\omega_{\iota }}y\right)q_{1}+\left(\bar{\omega_{\iota }}z\right)r_{1}+\left(\bar{\omega_{\iota }}\right)s_{1}+\left(\bar{\omega_{2}}x\right)p_{2}+\left(\bar{\omega_{2}}y\right)q_{2}+\left(\bar{\omega_{2}}z\right)r_{2}+\left(\bar{\omega_{2}}\right)s_{2} \end{array} \end{array}$$

For training fuzzy system, ANFIS employs a back-propagation scheme for the parameters associated with the input membership functions, and least mean square estimation for the parameters associated with the output membership function [24]. The hybrid training algorithm is based on the following criteria: in the forward step of the hybrid algorithm, the outputs of the nodes will forward to the layer 4 and the consequence parameters are identified by the least squares method. In the backward step, the error signal is propagated backward and the premise parameters are updated by gradient descent method [30].

## 3. Subtractive Clustering

In order to optimize the fuzzy system and increase its ability for EMG pattern recognition, subtractive clustering was employed to optimize fuzzy rules specification. This method partitions the data into groups (clusters), and generates a Fuzzy Inference System (FIS) with the minimum number of rules required to distinguish the fuzzy qualities associated with each of the clusters. Therefore, the utilization of clustering algorithms allows characterization and organization of data, but also the construction of models from a database. Basically clustering divides data sets derived from a large group into similar groups. Clustering can be used to model an initial fuzzy network, in other words, to determine the fuzzy rules. For this purpose, the clustering technique is validated based on the following propositions: (a) similar entries in a target system should be modeled to produce similar outputs and (b) these similar pairs input-output are packed in clusters of the training data set [30].

Subtractive clustering is based on a measure of the density of data points in the feature space. The idea behind this approach is to find regions in the feature space with high densities of data points. The point with the highest number of neighbours is selected as the center for a cluster. The data points within a prespecified fuzzy radius are then removed (subtracted), and the algorithm looks for a new point with the highest number of neighbours. This continues until all data points are examined [24].

The technique subtractive clustering proposed by Chiu [31], considers any data points as candidates for the cluster centers. Using this method, the processing is proportional to the number of data points, independent of the size of the problem under consideration. For example, is a collection of *n* data points {*x_1_*, …, *x_n_*} in an M-dimensional space, whose points were normalized to a hypercube. Since each data point is candidate for the cluster center, the density measurement at each point *x_i_* is defined by Equation (13):

(13) $${\text{D}}_{\text{i}}=\sum_{\text{j}=1}^{\text{n}}\text{exp}(-\frac{{\left\|{\text{x}}_{\text{i}}-{\text{x}}_{\text{j}}\right\|}^{2}}{{\left({\text{r}}_{\text{a}}\;/\;2\right)}^{2}})$$

where *r_a_* is a positive constant. A point will have a great density it has many neighbor points. The radius *r_a_* defines the neighborhood and the points outside of the neighborhood contribute very little to the density measurement. After the density measurement (*D_i_*) is calculated for all of the points, the point with highest density is selected to be the center of the first cluster. If *x_c1_* is the selected point and *D_c1_* your density value, the measured density for each point is revised according to the expression shown in Equation (14):

(14) $${\text{D}}_{\text{i}}={\text{D}}_{\text{i}}-{\text{D}}_{\text{c}1}\text{exp}(-\frac{{\left\|{\text{x}}_{\text{i}}-{\text{x}}_{\text{c}1}\right\|}^{2}}{{\left({\text{r}}_{\text{b}}\;/\;2\right)}^{2}})$$

After reviewing the density of each point, the next center *x_c2_* is selected and all of the density measures of the points are revised again. This process is repeated until a sufficient number of clusters are created. When applied the *subtractive clustering* technique for a set of input-output data, each cluster center will represents a prototype that exhibits certain characteristics of the system being modeled. This cluster centers are used as centers of the premises of the fuzzy rules in a zero order Sugeno model.

## 4. Experimental Methods

**Figure 3.**
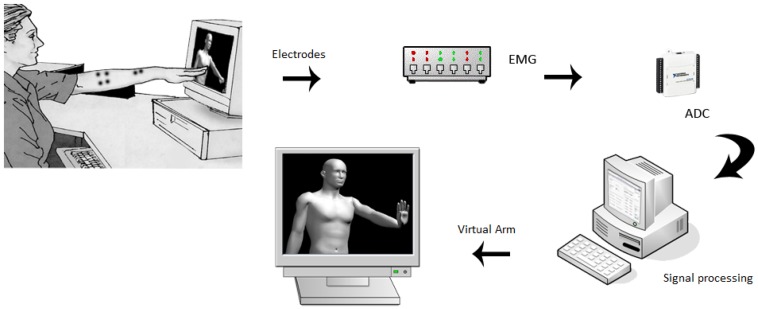
Block diagram of the proposed system.

**Figure 4.**
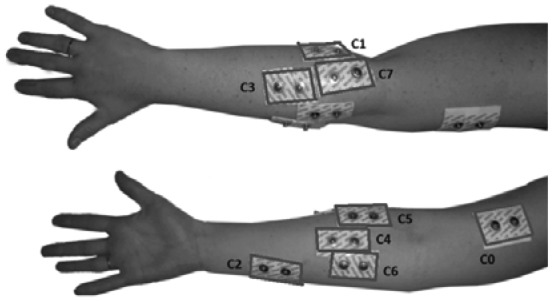
Picture showing the electrodes positions in the same arm (right arm).

The proposed experimental system (see Figure 3) consists of an LCD screen that generates visual stimulus with animations of random movements of the arm which should be replicated by the user. An 8-channel electromyography (EMG) system is used with surface electrodes placed in strategic places (see Figure 4 and Table 1) previously defined on the right arm to capture the myoelectric signal during displayed movements. The recording signal is performed using bipolar electrodes of passive configuration. Eight pairs of electrodes located in the main muscle groups (see Table 1) of the subject (only right arm) were been used, which are the main part of the movements that were chosen to characterize: *biceps* (*C_0_*), *palmaris longus* (*C_1_*), *flexor carpi ulnaris* (*C_2_*), *flexor carpi radialis* (*C_3_*), *pronator teres* (*C_4_*), *extensor digitorum* (*C_5_*), *brachio radialis* (*C_6_*) and *extensor carpi ulnaris* (*C_7_*), as shown in Figure 4. The experimental methodology is similar to the work of Winkler and Balbinot [32], but a larger number of tests has been performed and the volunteers are trained (accustomed to using this system).

The goal of preprocessing step is to prepare the signal for the sub sequent steps and reduce noise artifacts. Because of the very sensitive nature of EMG signals, they can be easily contaminated by different kinds of noise sources and artifacts which will contribute to very poor classification results. The noise was eliminated by using typical filtering procedures such as band-pass filter, band-stop filter and by use of a good quality of equipment with a proper electrode placement. The signal was amplified with a high common mode rejection ratio (CMRR) amplifier (which provides a high common mode rejection ratio of 110 dB). A notch filter (60 Hz) was used to eliminate power line noise. Through a data acquisition board (NI-USB6008 with an acquisition rate of 1 kHz per channel), the myoelectric signal is digitized and processed on a portable computer, where it is filtered (high-pass filter with 500 Hz cut-off frequency to reduce motion artifacts and a low pass filter of 20 Hz cut-off frequency to reduce noise, it was found that the dominant frequency was in the range of 70–300 Hz) and analyzed by software using the technique of pattern recognition, based on neuro-fuzzy systems. Finally, the system has as output the characterization of the movement and also verifies if the executed movement was well recognized.

**Figure 5.**
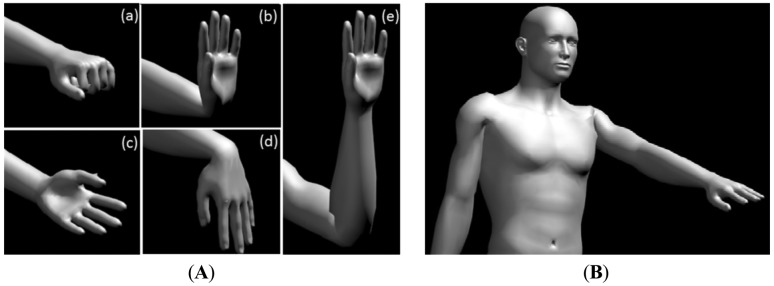
Details virtual model: (**A**) zoom of the hand: (**a**) hand contraction, (**b**) wrist extension, (**c**) forearm rotation, (**d**) wrist flexion, (**e**) forearm flexion e; (**B**) whole body model.

The virtual model created in this work aims to help the standardization of tests for the acquisition of the myoelectric signal. With this virtual model is possible, for the subject, visualize the movement to be performed during the tests, so that all subjects perform, as best as possible, the same movements at the same time base, making the system more user-friendly. For the development of the virtual model we used the software MakeHuman Alpha5 and Blender 1.0 Beta 2:54. Figure 5 shows details of the virtual model.

This virtual model is a skeleton whose manipulative joints are used to define the positions that it should take. For the development of the animation it was necessary to set the start and end position and movement timing of each of the respective movement. The software then builds an animation by connecting the two points during a defined duration. A rest position was also established which was adopted for all movements. All movements start from the rest position, run and return to it. To display the animations, a routine was developed enabling the reading of files and reproduction the virtual model.

**Figure 6.**
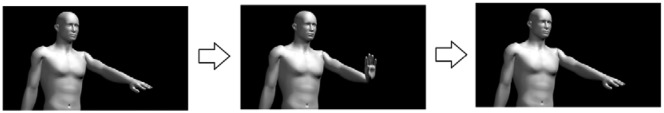
Static representation of a simple movement: wrist extension movement.

There are seven movements, which are: Wrist Flexion; Hand Contraction, Wrist Extension, Forearm Flexion, Forearm Rotation, Hand Adduction and Hand Abduction (for example see Figure 6). For the movements the following time sequence with a total duration of each animation of 8.3 s was adopted: initial interval: 0.4 s in which the animation will be in the rest position; forward movement: duration of 2.9 s; movement interval: 1.25 s, in which the animation remains static at the end of the ongoing movement; backward movement: same duration as the forward movement (2.9 s); final interval: duration of 0.8 s, during which the animation is again in the rest position.

The data was collected from thirty able-bodied subjects. All the experiments were carried out with consent of the subjects, according to the ethical precepts. In order to standardize the testing of signal acquisition the following aspects were considered: each test consists of five sessions; a random sequence of animations is generated for each session of the test; each session is composed of five repetitions of each of the seven selected movements; between movements, the subject should rest for 3 s and each subject participates in a single test (the subjects were instructed to relax between the movements and maintain each gesture comfortably). There were no restrictions or measurement of the force exerted by the subjects during arm movements.

To start the acquisition, after correct positioning of the electrodes, the subject is instructed to replicate the animations of the virtual model, which appear on the LCD screen. The classification of each movement occurred during the data acquisition. A routine was developed to generate the sequence of movements randomly. The output is a vector with a random order of the movements of the virtual model presented to the user. The neuro-fuzzy system takes as input the rms values of each pre-processed data acquisition channel (calibration of each channel, removal of DC component, filtering, windowing the signal of interest and determining the rms value of the signal of interest).

The system presents as output the characterized movements that are being carried out by the human arm. The fuzzy-neural network is interfaced via Labview, where the routine developed in Matlab is called when needed, being processed in the background. First the number of network inputs, which can vary from 2 to 8, depending on the number of channels which is intended to analyze, was set. The channel that will be used on the network can be selected by the operator of the system in which the developed routine performs reading of all channels and automatically separates the desired channels for processing. This function has as input the array of channels to be selected and as output only the desired channels. The output of the neuro-fuzzy networks is considered fixed, containing the 7 movements previously determined. The output values ranges from 0 to 1, and for each movement there is a corresponding fixed known value, as shown in Table 2.

The developed structure is a fuzzy network type Sugeno obtained in the generation of an initial structure adapted from an input-output set acquired in the systems tests. The structure contains eight inputs and one output. To adjust the system it is necessary first to create an initial fuzzy network, which should be representative of the subject data. For this, the subtractive clustering technique was used, which can generate, from a input-output data, membership functions of input and output, and the fuzzy rules structure for type Sugeno. This technique was chosen because it obtained good results in preliminary studies cases. In the first subject assay, the expected input and output values are used to create the system initial structure representing the fuzzy network of eight inputs, 60 clusters (*i.e.*, 60 rules) and one output, generated for a system assay, and adjust it later to adapt it to represent more faithfully a model that can characterize the subject movements.

After creating the initial fuzzy structure is necessary to adapt the membership functions for the data acquired in the session, thus making a fine adjustment of the functions, leading to results more consistent with the ones expected. The adaptation step is very important, because it helps to better define the limits and parameters of the membership functions, leaving the model best suited for the subject. In this step a hybrid training function was used. The hybrid training is a combination of the gradient method with the LSE method to optimize the time convergence of the model, since it reduces the demand on the dimensional space. As output of the training step, a fuzzy network with adapted membership functions to a particular subject is generated, causing the limits of each function to be left according to the training data.

## 5. Results and Discussion

This topic will discuss the tests performed during system development, and the results obtained. It is important to note that the pre-processing routine and calibration have already been validated in previous studies [7,8]. Subjects participating in this research presented an age range of 20 ± 5 years of age, and were of both sexes. Altogether trials were conducted with thirty subjects. The abbreviations of the characterized movements are presented in Table 2. It is worth noting that the parameters of ANFIS training were the same for all subjects.

**Figure 7.**
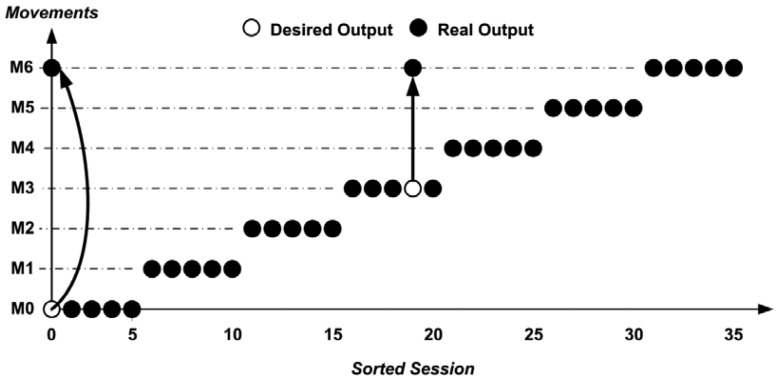
System output for Subject 1–Section 3 (5 repetitions).

For example, Figure 7 represents the result of section 3 for Subject 1 where is possible to notice that for the movements M1, M2, M4, M5 and M6 an accuracy of 100% is obtained. Movements in which an incorrect recognition occurred, for example, were the M3 (forearm flexion) with M4 (forearm rotation), causing 20% error, which may occur since these movement uses common muscles, such as the biceps, and only surface electrodes were used. The first movement M0 (hand contraction) was considered the M6 (hand adduction), because it was wrongly executed by the user (check during this test).

**Figure 8.**
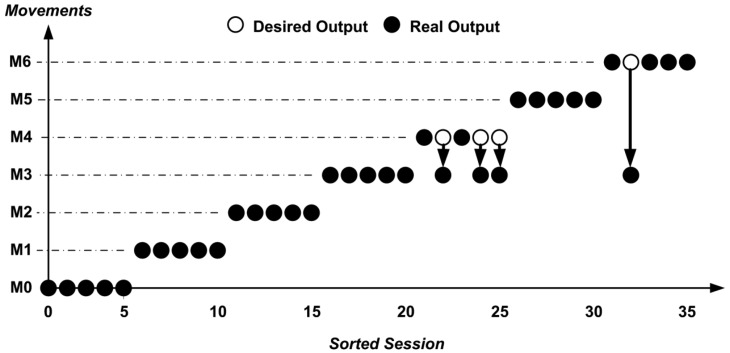
System output for Subject 27–Section 2 (5 repetitions).

Figure 8 represents the result of Section 2 for Subject 27 where is possible to notice that for the movements M0, M1, M2, M3 and M5 an accuracy of 100% was obtained. Movements in which incorrect recognition occurred were the M4 (forearm rotation) with M3 (forearm flexion), causing a 60% error, which may occur since these movements uses common muscles, such as the biceps, and only surface electrodes were used. The second repetition of movement M6 (hand adduction) was recognized as M3 (forearm flexion) because the user made the motion requested by half (check during this test).

For example, Table 3 represents the average accuracy rate of the system for each movement per session, and the overall average of each movement per session for Subject 1. The movements with lower hit rate are: forearm flexion (M3), forearm rotation (M4) and hand adduction (M6), with 85%, 90% and 90% hit rate, respectively. This is due to the similarity between M3 and M4 and the difficulty of the subject in performing the movement M6.

**Figure 9.**
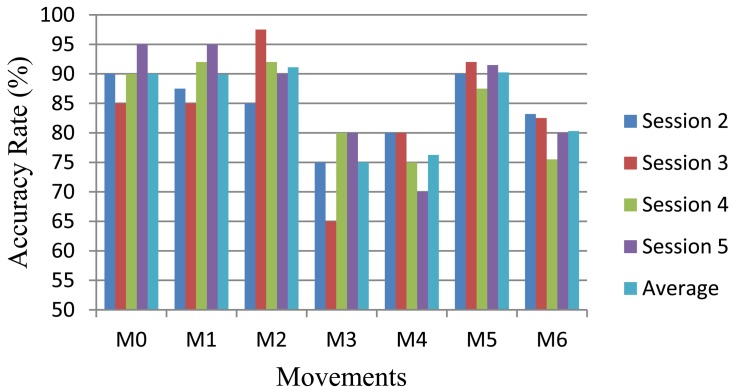
Overall result of the system for each movement.

Figure 9 represents the average of all tests performed for each movement. Analyzing the graph it is clear that the more accurate movements were M0, M1, M2, M5, with averages rates of approximately 90%. These movements are quite distinct, which increases the accuracy rate of the system. Overall the system achieved an average accuracy of 86%.

As it was possible to see from the results of this work, most of the errors were caused by similar movements, or the differentiation of compound movements with their simple movements. The human arm has many degrees of freedom and is able to develop a system that can characterize many different movements and combined them which is the real challenge and for this reason is an active area of research [7,8].

However, for easy observation observed and to confirm the investigation, analysis of variance (ANOVA) and the multiple comparisons were used (therefore, for statistical validation methodology was used the design and analysis of three-factor experiments—Three-Factor Fixed Effects Model). The ANOVA provides a statistical test of whether or not the means of several groups are all equal. If there are not all the same, you may need information about which pairs of means are significantly different, and which are not. A multiple comparison procedure is a test that can provide such information. Two means are significantly different if their intervals are disjoint, and are not significantly different if their intervals overlap. This experimental design is a completely randomized design. Consider the three-factor-factorial experiment, with underlying model Equation (15):

(15) $$\;Y_{\text{ijkl}}=\mu +\tau_{i}+\beta_{j}+\gamma_{k}+{\left(\tau \beta \right)}_{ij}+{\left(\tau \gamma \right)}_{ik}+{\left(\beta \gamma \right)}_{jk}+{\left(\tau \beta \gamma \right)}_{\text{ijk}}+{∊}_{\text{ijkl}}\{\begin{array}{c} i=1,2,\ldots ,a\\ j=1,2,\ldots ,b\\ k=1,2,\ldots ,c\\ l=1,2,\ldots ,n \end{array}$$

where *μ* is the overall mean effect, *τ_i_* is the effect of the *i*th level of factor *A* (eight different muscles or eight channels 0–7), *β_j_* is the effect of the *j*th level of factor *B* (thirty subjects: 1 and 30), *γ_k_* is the effect of the *k*th level of factor *C* (seven movements: hand contraction, wrist extension, wrist flexion, forearm flexion, forearm rotation, hand abduction and hand adduction), (*τβ*)*_ij_* is the effect of the interaction between *A* and *B*, (*τγ*)*_ik_* is the effect of the interaction between *A* and *C*, (*βγ*)*_jk_* is the effect of the interaction between *B* and *C*, (*τβγ*)*_ikj_* is the effect of the interaction between *A*, *B* and *C* and *∈_ijkl_* is a random error component having a normal distribution with mean zero and variance σ^2^ Notice that the model contains three main effects (*A*,*B* and *C*), three two-factor interactions, a three-factor interaction, and an error term.

The *F*-test on main effects and interactions follows directly from the expected mean squares. These ratios follow *F* distributions under the respective null hypotheses. We will use *α* = 0.05 (significance level). The analysis of variance for a three-factor experiment showed that the main effects due to the eight channels, thirty subjects and seven movements are significant, in other words, there is a strong evidence to conclude that *H*_0_ is not true (the variances of the three main effects are different). Thus, it is possible to say that the output rms for each one of the eight channels, thirty subjects and seven movements are quite distinct from each other, and thus, the myoelectric signals are also distinct and so can be treated as distinct channels by the developed neuro-fuzzy system. The results of this model showed that the interactions are true, *i.e.*, (*τβ*), (*τγ*), (*βγ*) and (*τβγ*) are significant. Table 4 presents several studies with similar characteristics: mathematical method, movements' number or objective of the study.

The results indicated that the ANFIS system exhibits similar performance compared to other studies (see Table 4). The system developed by Chan using fuzzy techniques classified four simple movements with an accuracy of 91% [5]. A system was developed by Ajiboye to characterize four classes of movements using four channels, obtaining an accuracy of 86% [4]. These systems had similar results to those found in the preliminary study of this research in which the neuro-fuzzy technique was used to classify five distinct movements using three signal acquisition channels, obtaining an accuracy of 86% [7,8]. Another difference that it is important to note is that the proposed study used only one feature extracted for each channel (RMS value), without feedback, without extraction of channels or data, unlike other studies that use many features per channel and many techniques for classification. The results found in this study when compared to the work [32] demonstrate the importance of training the volunteers. In this study, the volunteers used the system for at least 3 months and thus had a higher hit rate.

## 6. Conclusions

The proposed system was designed to use a limited amount of up to eight channels of myoelectric signal acquisition and with the assistance of a more robust artificial intelligence technique was able to verify the validity of this system in terms of performance in the characterization of seven distinct movements. With the windowing signal occuring at the instant when a movement occurs, it is possible to obtain the rms value for each of the eight channels and to use these values as input to a neuro-fuzzy network with one output an up to eight inputs. This network aims to characterize the movements that are being executed. The network is adapted in accordance with supervised training, to evaluate system performance over time. As can be seen on the results, some movements achieved a lower hit rate, this may occur due to poor signal quality, user error, and the amount of motion that was presented to the neuro-fuzzy network, since most of the errors were caused by similar movements, or the differentiation of compound movements with their simple movements, which have a very similar rms value response, causing the network to get confused. The average hit rate accuracy obtained was 86% for seven distinct movements in tests of long duration, about three hours.

## Figures and Tables

**Table 1.** Movement defined for each channel.

**#Channel**	**Muscle**	**Main Movement**
C_0_	*Biceps*	Forearm Flexion
C_1_	*Flexor Carpi Ulnaris*	Hand Abduction
C_2_	*Flexor Carpi Radialis*	Hand Adduction
C_3_	*Extensor Digitorum*	Hand Contraction
C_4_	*Pronator Teres*	Forearm Rotation
C_5_	*Brachioradialis*	Forearm Rotation
C_6_	*Palmaris Longus*	Wrist Flexion
C_7_	*Extensor Carpi Ulnaris*	Wrist Extension

**Table 2.** Network output values associated with the recognized movements.

**Movement**	**Corresponded Output**	**Abbreviation**
Hand Contraction	0	M0
Wrist Extension	0.083	M1
Wrist Flexion	0.166	M2
Forearm Flexion	0.249	M3
Forearm Rotation	0.333	M4
Hand Abduction	0.416	M5
Hand Adduction	0.499	M6

**Table 3.** Summary of the system average accuracy rate to the Subject 1.

**Subject 1**	**M0**	**M1**	**M2**	**M3**	**M4**	**M5**	**M6**
Session 2 (%)	100	100	100	100	80	100	80
Session 3 (%)	80	100	100	80	100	100	100
Session 4 (%)	100	80	100	80	100	80	100
Session 5 (%)	100	100	100	80	80	100	80
Average (%)	95	95	100	85	90	95	90

**Table 4.** Results from other studies.

**Selected Study**	**Ahsan *et al*. [16]**	**Rajesh *et al*. [18]**	**George *et al*. [25]**	**Shenoy *et al*. [19]**
Movements used	Left, right, up and down	Hand extension, hand grasp, wrist extension, wrist flexion, pinch and thumb flexion	Classification of different speeds of movement of human elbow	Static hand gesture (gestures correspond to pairs of actions: grasp-release, left-right, up-down and rotate)
Features	Mean absolute value, RMS, variance, standard deviation, zero crossing, slope sign change and Wilson amplitude	Entropy, RMS and standard deviation	Mean absolute value and variance	RMS value
Classification	Artificial neural network	Euclidean distance	Fuzzy Logic Classifier (FLC) and Probabilistic Neural Network Classifier (PNNC)	Linear Support Vector Machines
Hit Ratio	Average of 88.4%	For feature RMS was 83.33%	97.3% for FLC and 93.6% for PNNC	Accuracy of 92 to 98%
**Selected Study**	**Kurzynski *et al*. [24]**	**Khezri *et al*. [27]**	**Khezri *et al*. [26]**	**Boschmann *et al*. [20]**
Movements used	Seven elementary actions were distinguished in the process of grasping with a hand: rest position, grasp preparation, grasp closing, grabbing, maintaining the grasp, releasing the grasp, transition to the rest position	Eight hand movements: hand opening and closing, pinch, thumb flexion, wrist radial flexion and extension and wrist flexion and extension.	Hand motion commands (hand opening and closing, pinch and thumb flexion, wrist flexion and extension), but with vision feedback to increase the capability of the system	Seven different movements: extension, flexion, ulnar deviation, radial deviation, pronation, supination, open, close, key grip, pincer grip and extract the index finger
Features	Six types of grapes depending on the grasping object (a pen, a credit card, a computer mouse, a cell phone, a kettle and a tube)	Time domain, time-frequency domain and their combination	Mean absolute value, slope sign changes and AR model coefficients	
Classification	Five types: Bayes approach with Markov model, multilayer perceptron, multiclassifier with competence function, classifier based on fuzzy logic and classifier based on Dempster-Shafer theory of evidence	Fuzzy inference system (FIS) and Artificial neural network (ANN)	Adaptive neuro-fuzzy inference system (ANFIS)	Support vector machines
Hit Ratio	Mandani inference system is applied with the one-instant-backwards and the two-instant-backwards dependence (algorithms FS1 and FS2): the classification accuracies of sequential classifiers compared in the experiment: for FS1: 72.5 (order of AR model was 2) to	Average accuracy for eight movements was of the 83% to 78% (the best combination to design sEMG pattern recognition system)	Average results of the neuro-fuzzy system: opening-98%; closing-100%; wrist flexion-94%; wrist extension-96%; pinch-98%;	Accuracy averaged over all 11 movements is 91.3%
	89.7 (order of AR model was 8) and FS2: 69.5 (order of AR model was 2) to 88.5 (order of AR model was 8)		Thumb flexion-94% and average for six movements-96.67%	
Selected Study	This work			
Movements used	Seven movements: Wrist Flexion; Hand Contraction, Wrist Extension, Forearm Flexion, Forearm Rotation, Hand Adduction and Hand Abduction			
Features	RMS value			
Classification	Neuro-Fuzzy			
Hit Ratio	Average accuracy of 86%; Average accuracy of approximately 90% (hand contraction, wrist extension, wrist flexion and hand abduction)			
